# Short-Term Effects of Supplemental L-Arginine, Diosmin, Troxerutin, and Hesperidin in Diabetic Patients: A Pilot Study

**DOI:** 10.1155/2021/3508281

**Published:** 2021-12-02

**Authors:** Marco Bagnati, Chiara Puricelli, Giulia Bauce, Matteo Basile, Barbara Grigollo, Flavia Prodam, Umberto Dianzani, Giorgio Bellomo, Roberta Rolla

**Affiliations:** ^1^SCDU Clinical Chemistry Laboratory, Maggiore della Carità University Hospital, Novara, Italy; ^2^Department of Health Sciences, Amedeo Avogadro University of Eastern Piedmont, Novara, Italy; ^3^SCDU Endocrinology, Maggiore della Carità University Hospital-Department of Translational Medicine, Amedeo Avogadro University of Eastern Piedmont, Novara, Italy

## Abstract

**Background and Aims:**

Inflammatory, oxidative stress, and endothelial dysfunction play a key role in the pathogenesis of long-term cardiovascular complications in patients with diabetes. The present observational prospective study is aimed at evaluating the effects of micronutrients and phytochemicals contained in the dietary supplement Flebotrofine® (AMNOL Chimica Biologica) on biochemical markers of inflammation, endothelial dysfunction, and glycemic control in patients with diabetes.

**Methods:**

105 type 1 or type 2 diabetes patients regularly took a daily dose of the dietary supplement Flebotrofine® for three consecutive months, and haematological and biochemical parameters were checked at baseline, after three months of treatment, and one month after its suspension. Statistical comparison of the laboratory parameters was performed using the two-tailed ANOVA test for repeated samples with a statistical significance level set at *p* < 0.05.

**Results:**

The daily use of Flebotrofine® did not change the glycemic metabolic compensation of enrolled patients. After three months of regular Flebotrofine® intake, the plasma levels of the antioxidant *β*-carotene and of arginine were significantly higher compared with the baseline values, with a decrease in the ADMA/arginine ratio. In contrast, apolipoprotein B, ApoB/ApoA1 ratio, and platelet and leukocyte counts significantly dropped.

**Conclusion:**

The daily use of Flebotrofine® might be a valid supplement of arginine, the precursor of NO, and essential in the prevention of endothelial dysfunction. The regular intake of arginine and phytochemicals also improved the antioxidant and antithrombotic profile of enrolled patients. Therefore, Flebotrofine® could be a useful dietary supplement to prevent long-term complications in patients with diabetes.

## 1. Introduction

Diabetes remains among the top 10 causes of death worldwide, according to the latest report of the World Health Organization (WHO) conducted in the last 20 years [1]. Recent studies have demonstrated that early endothelial dysfunction is an important component in the pathogenesis of diabetic micro- and macroangiopathy. The advent of insulin replacement therapy in the early 1900s and the increased efforts toward metabolic control of diabetes mellitus (DM) through oral hypoglycemic agents have certainly improved the life quality and expectancy of subjects with diabetes. On the other hand, long-term complications of this disease have become apparent, primarily related to the vascular system [2]. Lots of studies have demonstrated the role of micronutrients and phytochemicals, such as L-arginine (L-Arg), flavonoids, and antioxidants, in preserving endothelial function [3, 4].

In the literature, there are numerous studies on arginine and its possible role in improving the body's ability to metabolize glucose, promoting the secretion of insulin [5]. Moreover, L-Arg is the precursor of nitric oxide (NO), whose importance in maintaining endothelial function is well known [6]. L-Arg has also been shown to be an important agent against some chronic diseases [3]. Clinical studies have demonstrated that L-Arg supplementation increases antioxidant enzyme activities in patients with ischemic heart disease [7] and improves endothelial function and protection against oxidative stress in patients with type 2 diabetes [8]. Experimental studies have also shown that L-Arg treatment attenuates both renal injury and hypertension in rats and in the offspring of diabetic rats. After L-Arg administration, blood pressure levels and vascular reactivity return to normal [9], and inflammation is reduced in both plasma and kidneys [10]. Besides, L-Arg plays an important role in protecting the pancreas against oxidative stress and is a well-known insulin secretagogue [11, 12]. Many authors even hypothesize its function in regulating postprandial glucose, by stimulating the secretion of insulin and glucagon-like peptide-1 (GLP-1), an intestinal hormone that plays an important role in appetite regulation [13]. Finally, currently available data prove that oral supplementation with L-Arg can affect endothelium-mediated vascular functions [14–16].

To date, it is well known that the endothelium is not simply a covering layer of the vascular wall, but a dynamic organ with autocrine and paracrine hormonal functions, able to actively interplay with leukocytes and platelets. Overall, the endothelium is capable of producing both parietal effects, by regulating vascular tone and vascular remodeling and luminal effects, by modulating hemostasis, and by interacting with leukocytes. All these functions are accomplished thanks to plenty of molecular mediators synthesized and released by endothelial cells. Among these, the vasodilating ones, such as NO or prostaglandins, also possess antithrombotic, antiproliferative, and antiatherogenic properties, while vasoconstrictors like endothelin lead to opposite effects.

**Figure 1:**
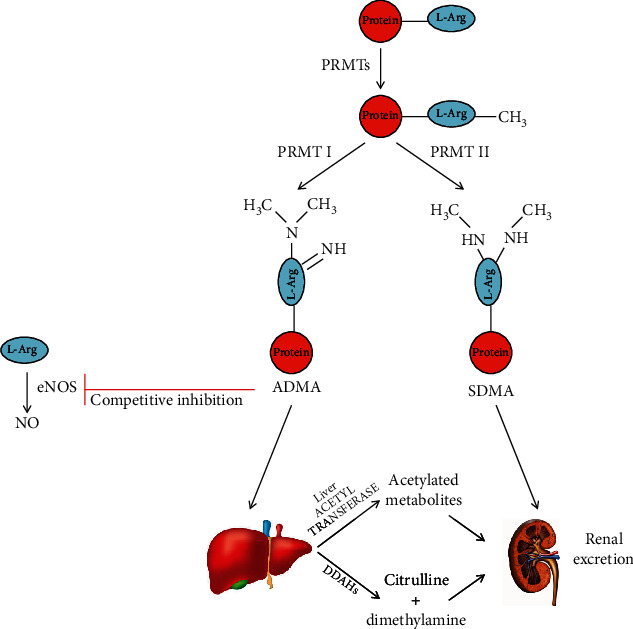
Graphical representation of the metabolic pathways and functions of N^G^,N^G^,dimethyl-L-arginine (ADMA). ADMA: N^G^,N^G^,dimethyl-L-arginine; DDAH: dimethylarginine dimethylaminohydrolase; eNOS: endothelial nitric oxide synthase; L-Arg: L-arginine; NO: nitric oxide; PRMT: protein arginine methyltransferase; SDMA: symmetric dimethylarginine.

In this scenario, the endogenous endothelial nitric oxide synthase (eNOS) inhibitor N^G^,N^G^,dimethyl-L-arginine (ADMA) has gained interest as a potential marker of endothelial dysfunction positively correlated with risk factors for atherosclerosis and cardiovascular disease (CVD) under a broad range of circumstances [17–19]. ADMA, together with its inactive stereoisomer symmetric dimethylarginine (SDMA) and monomethylarginine (MMA), is synthesized posttranslationally from L-Arg methylation by protein arginine methyltransferases (PRMTs), and it competes with the NO precursor L-Arg, thus limiting NO production [20, 21]. While SDMA is entirely eliminated by renal clearance, ADMA is first catabolized to citrulline by dimethylarginine dimethylaminohydrolase (DDAH), expressed in the liver, pancreas, kidneys, and endothelium, before being excreted in the urine, and it can be considered the predominant eNOS inhibitor (Figure 1) [20].

Finally, a key role in the long-term complications of patients with diabetes is played by hyperglycemia, responsible for oxidative stress, which in turn favors oxidation and nonenzymatic protein glycation, in particular of low-density lipoproteins (LDL), generating advanced glycation end-products (AGEs). As a consequence, the endothelium loses its antithrombotic properties, resulting in monocyte adhesion and infiltration and early atherogenesis. Interestingly, it seems that the oxidative damage already affects the microcirculation at the endothelial level even before macroangiopathy develops, providing an example of a pathological continuum. The most apparent alteration is a thickening of the basement membrane, which, in the long term, may lead to microvessel loss and pruning. This neovascularization ultimately worsens atherogenesis in larger vessels, likely through a mechanism also involving a hypoxia-ischemia cycle affecting the *vasa vasorum* [22].

The present study is aimed at evaluating the effects of dietary supplements on biochemical markers of inflammatory and oxidative stress, endothelial dysfunction, and glycemic control in patients with diabetes. More specifically, the study focused on the dietary supplement Flebotrofine®, produced by Amnol Chimica Biologica (AMNOL Chimica Biologica S.r.l., Novara, Italy) to investigate whether dietary integration with micronutrients could be a beneficial adjunct to the traditional treatment in patients with diabetes to improve their cardiovascular profile.

## 2. Materials and Methods

### 2.1. Study Design

105 patients with diabetes referring to the Diabetes Center of the Endocrinology Unit of Maggiore della Carità University Hospital (Novara, Italy) were enrolled and asked to take a daily dose of the dietary supplement Flebotrofine® for three consecutive months. Flebotrofine® is available as a soluble powder and contains L-Arg, micronized diosmin, troxerutin, hesperidin, and plant extracts, including those from black currant and *Centella asiatica* (Table 1). L-Arg is known for its vasodilating activity, while diosmin, troxerutin, and hesperidin belong to the flavonoid family and exert antioxidant, antiapoptotic, anti-inflammatory, and vasoprotective functions [4]. Additionally, their role in modulating glucose metabolism by affecting its uptake and storage and peripheral insulin resistance has been described by several *in vivo* studies [23–25]. Black currant is known to improve lymphatic drainage and to act as an antioxidant [26], while *Centella asiatica* contains glycosidic triterpenoids able to modulate NO and collagen production and has antioxidant, antiapoptotic, and anti-inflammatory properties [27].

After receiving a full description of the research project, all participants gave their informed written consent to be enrolled in the study, to follow the planned intervention, and to show up at each scheduled appointment so that their consent was considered valid for the entire duration of the study. All procedures were approved by the Italian ethical committees (study name: FLEBO: CE 23/16) according to the Helsinki Declaration.

The inclusion criteria were a diagnosis of type 1 or type 2 diabetes and age between 40 and 85. Exclusion criteria were acute and chronic hepatic disease and renal failure.

Supplementation compliance was monitored thanks to scheduled appointments with the subjects enrolled every 3 weeks. During those meetings, a sufficient quantity of Flebotrofine® was provided for the following 21 days.

Blood samples were collected at baseline, after three months of treatment, and one month after its suspension. At each time point, hematological and biochemical parameters were evaluated, with a specific focus on inflammatory stress, endothelial dysfunction, and glycemic control, for a total of 54 analytes tested.

A single-arm study has been used to evaluate Flebotrofine® efficacy due to the great difficulty encountered in enrolling a healthy control group comparable for age and sex. However, the data obtained after three months of Flebotrofine® supplementation were compared both with the data at zero time and with those obtained after one month of Flebotrofine® washout.

### 2.2. Demographic and Clinical Characteristics of the Study Participants

133 subjects with diabetes initially agreed to participate. However, 8 of them did not show up at the first appointment so that the subjects enrolled were 125. Of these, 20 were lost to follow-up, for a total of 28 patients since the beginning of the study, with a drop-out rate of 21.1%. The number of patients who completed the whole study was thus 105.

Among the 105 subjects enrolled, 65 were males (61.9%), and 40 were females (38.1%). The mean age considering both categories was 59.1 (59.6 for males, 58.5 for females). 11 patients had type 1 DM (10.5%), while the majority (94) had a diagnosis of type 2 DM (89.5%). Regarding drug treatment, 46 subjects were taking only oral hypoglycemic agents (43.8%), 27 were under insulin-replacement therapy (25.7%), 25 received a combined treatment (23.8%), and seven were following only diet therapy (6.7%).

### 2.3. Biomarker Analysis

Laboratory measurements were performed in the Clinical Biochemistry Laboratory, Department of Health Sciences, Amedeo Avogadro University of Eastern Piedmont, Novara, Italy. Inflammatory, oxidative stress, and endothelial dysfunction blood markers were evaluated before treatment and twice thereafter, as described above.

High-sensitivity C-reactive Protein (hs-CRP), cholesterol, triglycerides, high-density lipoproteins (HDL), low-density lipoproteins (LDL), apolipoprotein A (ApoA), ApoB, and GPT/ALT were tested in heparinized plasma on the ADVIA® 1800 Clinical Chemistry System or ADVIA® Chemistry XPT System (Siemens Healthcare GmbH, Erlangen, Germany). Hs-CRP was tested using an immunoturbidimetric method optimized with latex particles [28]. Triglycerides, cholesterol, HDL, and LDL were measured with an immunoenzymatic method with a final colorimetric Trinder reaction [29, 30]. For ApoA and ApoB, a polyethylene glycole- (PEG-) enhanced immunoturbidimetric method was employed [31]. ALT was determined through a kinetic enzymatic reaction, as described by Bergmeyer et al. [32].

L-Arg and ADMA were analyzed by implementing a new protocol based on the high-performance liquid chromatography/tandem mass spectrometry (HPLC-MS/MS) technique [33–35], through a manual procedure comprising protein precipitation by addition of ethanol, followed by an evaporation step with nitrogen. The residue was derivatized into butyl ester with n-butanol and HCl (Fisher's esterification) at 65°C for 15 minutes, then newly evaporated with nitrogen and reconstituted with a mixture of mobile phase and methanol. The analysis was performed using the HPLC-MS/MS technique with isotopic dilution by the Agilent 1290 Infinity II LC System and the Agilent 6410 Triple Quadrupole LC/MS mass spectrometer (Agilent, Santa Clara, CA, USA). Separation conditions were in gradient elution (HCOONH_4_/HCOOH in water as mobile phase A and HCOOH in acetonitrile as mobile phase B) with a reverse-phase column and multiple reaction monitoring.

Vitamin A and *β*-carotene levels were assessed after liquid-liquid extraction with hexane followed by injection in HPLC and analysis in isocratic conditions using a mobile phase (acetonitrile/dichloromethane/methanol) with UV detection at 460 nm. The HPLC system used was the Agilent 1260 Infinity II LC System equipped with a diode array detector (Agilent, Santa Clara, CA, USA) [36].

To test the flavonoids contained in Flebotrofine®, a new analytical method was implemented using an HPLC technique followed by mass spectrometry [37]. Hesperidin, diosmin, and rutin are metabolized to aglycone by intestinal bacteria, which separate them from their disaccharide-glycone, absorbed in this form and excreted as glucuronyl-derivatives in the urine. As a result, the blood concentrations of the aglycones hesperetin, diosmetin, and quercetin were evaluated, respectively. Serum samples were analyzed with and without hydrolysis, and the latter was performed either enzymatically, using *Helix pomatia* glucuronidase and incubating at 55°C for 30 minutes, or through the addition of KOH with incubation at 70°C for 30 minutes, to avoid the possible interference of flavonols in the glucuronidase used. After hydrolysis, the solution was brought to a pH of 2.5 to allow flavonol extraction in an organic solvent with HCl. After the addition of methyl-tert-butyl-ether and centrifugation, the supernatant was evaporated with nitrogen. The residue was reconstituted with methanol and water and analyzed by the Agilent 1290 Infinity II LC System and the Agilent 6410 Triple Quadrupole LC/MS mass spectrometer (Agilent, Santa Clara, CA, USA). However, it was not possible to implement a new protocol for troxerutin quantification since the methods described in the literature suggest a dedicated method which was not feasible in our laboratory [38, 39].

Finally, a complete blood count (including red blood cells, leukocytes, leukocyte differential count, and platelets) was performed on whole blood in K_2_-ethylenediaminetetraacetic acid (EDTA) anticoagulant on the XN-2000™ Hematology System (Sysmex Corporation, Kobe, Japan). Glycated hemoglobin (HbA1c) was measured in EDTA-whole blood by the ARIANT™ II System HbA1c Program (BioRad Laboratories, Hercules, CA, USA). For organizational reasons, it was not possible to measure the postprandial glycemic response at 2 hours, certainly an element of great interest for the study.

### 2.4. Statistical Analysis

Statistical analysis was performed on all hematological and biochemical parameters evaluated at three different time points using the analytical software SPSS v.15.0 (SPSS Inc., Chicago, IL, USA). The normal distribution of continuous variables was evaluated using the q-q plot, Kolmogorov-Smirnov, and Shapiro-Wilk tests, and all continuous variables were represented as mean and standard deviation (SD). The clinical characteristics of the participants and their laboratory parameters were compared using the Chi-Square and two-tailed paired ANOVA tests for categorical and continuous variables, respectively. Correlation analysis was performed using Spearman's correlation coefficient and Pearson's parametric coefficient, when appropriate. Statistical significance was calculated with the nonparametric Mann–Whitney test, using a *p* value < 0.05.

## 3. Results

The intake of Flebotrofine® for three consecutive months did not change the glycemic metabolic compensation of the patients enrolled, as confirmed by the values of HbA1c at baseline and after 3 months of dietary supplementation (7.26% vs. 7.15%, respectively).

HbA1c was measured instead of glycemia, not only because it is the ideal test to monitor the glycemic control of patients with diabetes but also because it allowed to evaluate their glucose plasma concentration during the three months of follow-up with a single final analysis.

**Figure 2:**
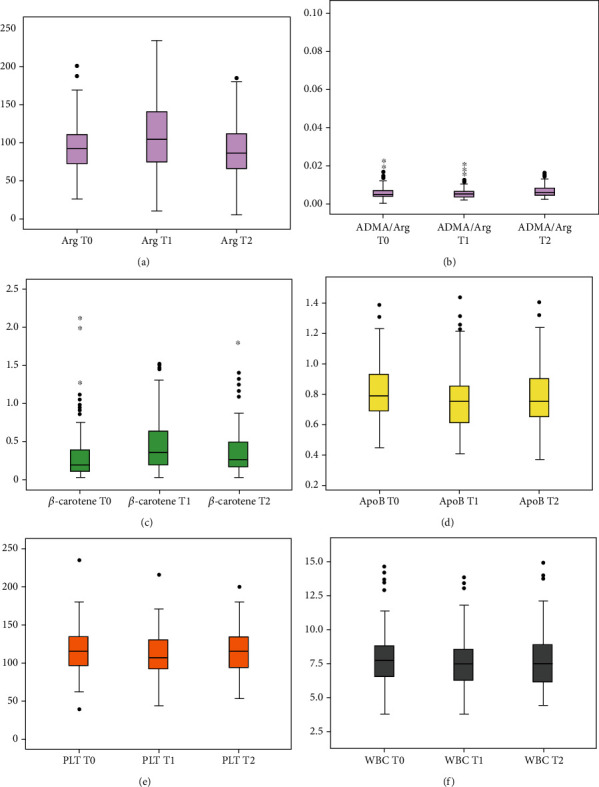
Box plots representing the distribution of parameter values at three different time points. Shown here are the value distributions of those parameters whose differences between the three chosen time points were the most statistically significant. The black horizontal bar inside the box graphically represents the median of the distribution of values (the 50th centile). The upper and lower borders of the box indicate the distribution quartiles or the 75th and the 25th centile, respectively. The lines extending above and below the boxes are the adjacent values, i.e., the most extreme observations that still do not extend more than 1.5 the height of the box beyond the quartiles and that contain about 99% of the observations. Finally, the black dots on one or on both sides of the boxes represent the atypical observations excluded from the majority of the recorded results. The time points analyzed were T0 (basal conditions), T1 (after three months of dietary supplementation with Flebotrofine®), and T2 (one month after treatment suspension): (a) box plots representing arginine values; (b) box plots representing the results of the ADMA/arginine ratio; (c) box plots displaying *β*-carotene value distributions; (d) box plots of apolipoprotein B (ApoB) results; (e) box plots representing platelet count distributions; (f) box plots showing the distributions of leukocytes.

The laboratory parameters measured at each time point are displayed in Table 2. The differences detected after three months of Flebotrofine® supplementation and one month after treatment suspension compared with baseline are highlighted, when statistically significant, reporting the statistical parameters calculated, including the *p* value. A graphical presentation of the same significant results can be found in Figure 2. L-Arg levels increased significantly after three months of Flebotrofine® intake compared with the baseline value (109.98 *μ*mol/L vs. 89.31 *μ*mol/L at baseline; *p* < 0.001). However, the amino acid plasma level dropped already one month after the suspension of dietary supplementation (90.89 *μ*mol/L). The ADMA/arginine ratio was reduced after three months of Flebotrofine® intake, but the decrease was not statistically significant (0.006 vs. 0.08 at baseline, *p* = 0.085).

*β*-Carotene levels increased significantly after three months of Flebotrofine® treatment (0.46 *μ*mol/L vs. 0.33 *μ*mol/L at baseline; *p* < 0.003). Furthermore, a reduction was observed already one month after the suspension of treatment (0.38 *μ*mol/L).

ApoB lipoprotein decreased significantly after three months of Flebotrofine® supplement (0.76 g/L vs. 0.81 g/L at baseline, *p* < 0.001) and remained lower even one month after the suspension (0.78 g/L), although statistical significance was lost. The ApoB/ApoA1 ratio decreased significantly after three months of supplementation and one month after the suspension of Flebotrofine® intake (0.60 and 0.60, respectively, vs. 0.63 at baseline, *p* < 0.01). On the other hand, the plasma levels of triglycerides, total cholesterol, LDL cholesterol, and HDL cholesterol remained unchanged after three months of Flebotrofine® supplement.

Regarding blood count parameters, platelet count decreased significantly after three months of supplement intake (237 × 10^9^/L vs. 227.3 × 10^9^/L at baseline; *p* < 0.001), whereas the white blood cell (WBC) count decreased significantly compared with the baseline value (7.60 × 10^9^/L vs. 7.89 × 10^9^/L; *p* = 0.01). As shown in Table 3, both reductions were significantly correlated (*p* < 0.001), and a correlation was also found with increased *β*-carotene plasma levels (*p* = 0.047 and *p* = 0.015, respectively).

Finally, the HPLC-MS/MS analysis of flavonoids was not able to dose their plasma concentration likely due to the rapid metabolism of these substances.

## 4. Discussion

The bulk of scientific literature about the pathophysiology of cardiovascular complications in diabetes has focused on vascular endothelial dysfunction as the *primum movens* of diabetic vasculopathy [40, 41]. Initially considered as a mere protective coverage of the vessel wall, the endothelium has been rediscovered as a multifaceted tissue with a complex physiology that plays key roles in blood homeostasis, ranging from blood flow dynamics to regulation of coagulation and inflammation, and with a potential implication in vascular disease.

An increasing number of scientific data show that micronutrient deficiency can contribute to endothelial dysfunction, which can be improved by the targeted administration of these substances [42, 43]. Among the micronutrients present in dietary supplements and potentially useful for the maintenance of microvascular trophism and the prevention of endothelial dysfunction, L-Arg, diosmin, troxerutin, hesperidin, and *Centella asiatica* extracts, contained in the Flebotrofine® dietary supplement considered in this study, are of particular interest.

Our data show how the daily use of the Flebotrofine® dietary supplement can be considered a valid strategy for L-Arg supplementation since the amino acid plasma levels are statistically higher after three months of Flebotrofine® intake. The main initial concern was the possibility of L-Arg to be converted into glucose, with a possible negative impact on glucose metabolism in a patient category already experiencing dysregulation of glycemic control. Nevertheless, the values of HbA1c observed before and after Flebotrofine® treatment demonstrated that the increase in L-Arg plasma level did not change the glycemic compensation in patients with diabetes [44]. Since L-Arg is a precursor of NO synthesis, its deficiency determines a reduced production of NO, threatening its protective effects of the intact endothelium. Indeed, NO is a ubiquitous mediator acting in the brain as a neurotransmitter, modulating the host immune response, and functioning as a vasodilator and endogenous antiatherogenic molecule in the cardiovascular system [45, 46]. The regulation of NO metabolism is particularly important in DM also because the activation of eNOS has been demonstrated to be under insulin control [47, 48]. The daily use of Flebotrofine® also decreased the ADMA/L-Arg ratio, although not significantly, by modulating the effect of ADMA, a competitive inhibitor of L-Arg conversion to NO by eNOS. Even previous studies observed that L-Arg supplementation restores the ADMA/L-Arg ratio to normal levels and thereby normalizes endothelial function [49].

Unfortunately, the HPLC-MS/MS analysis of flavonoids was not able to measure their plasma concentration. This is likely due to the very rapid metabolism of these molecules, which are eliminated in the urine as glucuronized metabolites already 12 hours after their intake. This aspect, together with a low quantity of flavonoids in Flebotrofine® (less than 1 g), suggests that serum levels of diosmetin, hesperetin, and quercetin are not detectable any more 24 hours after their intake when the second blood test was performed, even after a regular supplementation for three consecutive months. In this sense, it would be worth performing pharmacokinetic studies to better investigate the clearance and the variation of flavonoid concentration with time. Unfortunately, it was not possible to measure NO plasma levels and other markers of endothelial damage such as coagulation factor VIII (FVIII), von Willebrand factor (vWF), and vascular tone.

The daily implementation of the diet with Flebotrofine® in patients with diabetes led to a significant increase of *β*-carotene plasma levels, an important natural antioxidant, a precursor of vitamin A, essential to ensure the proper functioning of the circulatory, nervous, skeletal, and immune systems. In contrast, low plasma levels of *β*-carotene have been associated with a higher cardiovascular risk and a higher mortality rate [50]. Moreover, a statistically significant reduction in blood levels of ApoB and ApoB/ApoA ratio was observed after dietary supplementation with Flebotrofine® [51]. The ApoB/ApoA ratio is currently considered a better predictor of endothelial dysfunction, atherogenesis, and cardiovascular risk compared with cholesterol and triglycerides, especially in subjects with a normal or nearly normal lipid profile [51, 52].

It is important to note that the plasma levels of triglycerides, total cholesterol, LDL cholesterol, and HDL cholesterol remained unchanged after three months of Flebotrofine® intake. Therefore, rather than having an impact on the lipid profile by modifying the plasma lipid concentration, the Flebotrofine® supplement would seem to influence the lipoprotein composition, in particular by favoring the expression of an antiatherogenic lipoprotein phenotype [53].

Accordingly, the use of a supplement able to increase the plasma levels of L-Arg and *β*-carotene and to improve the lipid balance at the same time could be useful in patients with diabetes, exposed to a high risk of developing CVD in the long term. However, since the levels of L-Arg and *β*-carotene significantly dropped already one month after treatment suspension, it seems that this dietary supplementation should be regular and long-lasting, to ensure a constant level able to guarantee the efficacy of its properties.

Finally, the daily implementation of the diet with Flebotrofine® in patients with diabetes led to a significant reduction in the number of leukocytes and platelets, although not enough to expose the subject to infections or hemorrhagic risk, respectively. Platelets play a key role in the etiology and progression of atherosclerotic disease, being able to interact with endothelial cells and leukocytes, both by receptor-mediated contact and by the release of preformed or newly synthesized molecules with a vast array of functions, including pro-inflammatory and chemotactic effects. The recently coined process of thromboinflammation can thus provide an additional piece to the complex puzzle of atherogenesis [54–56]. Therefore, lowering the number of circulating platelets could indirectly lead to a decrease in cardiovascular risk.

Similarly, WBCs are involved in inflammatory responses and constantly interplay with the endothelium both in physiological and pathological conditions, contributing to the evolution of endothelial dysfunction and of the atherogenic plaque. Therefore, the daily implementation of the diet with Flebotrofine® could exert an anti-inflammatory effect, protecting patients with diabetes from oxidative damage and vessel wall inflammation thanks to direct modulation of the inflammatory response.

In conclusion, our results show that the daily use of Flebotrofine® can be considered a valid supplement of arginine, the precursor of NO, fundamental in the prevention of endothelial dysfunction. Moreover, the regular intake of L-Arg, diosmin, troxerutin, hesperidin, and plant extracts seems to improve the antioxidant and antithrombotic profile of enrolled patients.

Therefore, Flebotrofine® could be a useful support to the traditional therapy to prevent the long-term complications of diabetes; although to corroborate these conclusions, other studies are necessary to evaluate other markers of endothelial damage, such as NO, prostacyclin (PGI_2_), thromboxane A2 (TXA2), factor VIII (FVIII), von Willebrand factor (vWF), and the vascular tone.

## Figures and Tables

**Table 1:** Composition of the dietary supplement Flebotrofine®.

Component	Dose (mg)
L-Arginine	500
Troxerutin 95% of which troxerutin	315.8 300
Diosmin 80%—Microsmin plus of which diosmin	375 300
Black currant (extract)	200
Hesperidin 98% of which hesperidin	102.04 100
*Centella asiatica* 2% (extract)	50
Citric acid	750
Maltodextrin	1,513.16
Sucralose	11
Flavor	300
Fruitmax cranberry 712	140
Silicon dioxide	43
Total	4,300

ADMA: N^G^,N^G^,Dimethyl-L-arginine; DDAH: dimethylarginine dimethylaminohydrolase; eNOS: endothelial nitric oxide synthase; L-Arg: L-arginine; NO: nitric oxide; PRMT: protein arginine methyltransferase; SDMA: symmetric dimethylarginine.

**Table 2:** Comparison of the hematological and biochemical parameters evaluated at three time points in the 105 study participants.

Laboratory parameter	T0		T1		T2		T1-T0 difference			T2-T0 difference		
	Mean	SD	Mean	SD	Mean	SD	Mean	SD	*p* value	Mean	SD	*p* value
WBC (×10^9^/L)	7.89	2.09	7.60	1.93	7.70	2.13	-0.29	1.14	0.010^∗∗^			NS
RBC (×10^12^/L)	4.92	0.40	4.78	0.46	4.79	0.42			NS			NS
Hb (g/L)	144.68	13.53	141.36	13.69	140.94	13.42			NS			NS
HCT (%)	43.17	3.48	42.22	3.82	42.10	3.58			NS			NS
MCV (fL)	87.24	8.93	88.40	4.67	88.12	4.61			NS			NS
MCH (pg)	29.46	2.07	29.61	2.07	29.60	2.18			NS			NS
MCHC (g/L)	334.99	12.76	334.72	11.41	334.70	12.39			NS			NS
RDW-SD (fL)	42.54	3.08	42.89	2.79	42.67	3.09			NS			NS
RDW-CV (%)	13.28	0.87	13.33	0.93	13.30	0.89			NS			NS
PDW (%)	12.66	2.11	12.37	2.09	12.32	1.99			NS			NS
MPV (fL)	10.48	1.29	10.42	0.91	10.44	0.88			NS			NS
P-LCR (%)	29.87	7.50	28.60	7.30	30.82	22.76			NS			NS
PLT (×10^9^/L)	237.27	61.42	227.28	60.17	232.48	58.42	-9.99	22.52	<0.001^∗∗∗^	-4.79	23.13	0.036^∗^
HbA1_C_ (%)	7.26	1.16	7.15	1.07	7.16	1.10			NS			NS
HbA1_C_ (mmol/mol)	61.09	54.59	54.63	11.68	54.85	12.08			NS			NS
Creatinine (*μ*mol/L)	72.31	24.46	76.36	26.75	75.89	26.88			NS			NS
eGFR (mL/sec/m^2^)	0.88	0.23	0.87	0.20	0.87	0.19			NS			NS
Total cholesterol (mmol/L)	4.51	0.97	4.55	1.01	4.54	0.95			NS			NS
HDL cholesterol (mmol/L)	1.24	0.35	1.23	0.34	1.26	0.35			NS			NS
LDL cholesterol (mmol/L)	2.48	0.79	2.53	0.86	2.51	0.83			NS			NS
Triglycerides (mmol/L)	1.71	1.31	1.74	0.92	1.78	1.12			NS			NS
ApoA1 (g/L)	1.26	0.18	1.22	0.19	1.25	0.19	-0.04	0.15	0.013^∗^			NS
ApoB (g/L)	0.81	0.20	0.76	0.20	0.78	0.19	-0.05	0.12	<0.001^∗∗∗^	-0.03	0.14	0.020^∗^
ApoB/ApoA1ratio	0.63	0.15	0.60	0.14	0.60	0.15	-0.03	0.98	<0.01^∗∗^	-0.03	0.94	<0.01^∗∗^
GPT (ALT) (U/L)	32.06	24.24	28.70	14.91	29.10	15.41			NS			NS
hs-CRP (nmol/L)	36.70	43.77	33.62	39.94	37.50	46.40			NS			NS
Vitamin A (*μ*mol/L)	2.30	0.64	2.28	0.74	2.15	0.65			NS			NS
*β*-Carotene (*μ*mol/L)	0.33	0.36	0.46	0.37	0.38	0.34	+0.13	0.40	<0.01^∗∗^			NS
Arginine (*μ*mol/L)	89.31	35.17	109.98	47.06	90.89	38.02	+20.67	40.24	<0.001^∗∗∗^			NS
ADMA (*μ*mol/L)	0.45	0.11	0.52	0.11	0.51	0.10	+0.07	0.13	<0.001^∗∗∗^			NS
SDMA (*μ*mol/L)	0.73	0.30	0.80	0.29	0.79	0.24			NS			NS
ADMA/arginine ratio	0.008	0.012	0.006	0.005	0.008	0.011			NS			NS

^∗^, ^∗∗^, ^∗∗∗^Significance level (<0.05; <0.01; <0.001). NS: not significant; ADMA: N^G^,N^G^,dimethyl-L-arginine; ApoA1: apolipoprotein A1; ApoB: apolipoprotein B; eGFR: estimated glomerular filtration rate; GOT/AST: glutamate-oxaloacetate transaminase/aspartate aminotransferase; GPT/ALT: glutamate-pyruvate transaminase/alanine aminotransferase; Hb: hemoglobin; HbA1c: glycated hemoglobin; HCT: hematocrit; HDL: high-density lipoprotein; hs-CRP: high-sensitivity C-reactive protein; LDL: low-density lipoprotein; MCH: mean corpuscular hemoglobin; MCHC: mean corpuscular hemoglobin concentration; MCV: mean corpuscular volume; MPV: mean platelet volume; PDW: platelet distribution width; P-LCR: platelet large cell ratio; PLT: platelets; RBC: red blood cells; RDW-CV: red blood cell distribution width-coefficient of variation; RDW-SD: red blood cell distribution width-standard deviation; SDMA: symmetric dimethylarginine; T0: time point at baseline; T1: time point after three months of dietary supplementation with Flebotrofine®; T2: time point one month after treatment suspension; WBC: white blood cells.

**Table 3:** Correlations between the most significant variables evaluated in the 105 subjects under study after three months of dietary supplementation with Flebotrofine®.

	Arg T1	PLT T1	ApoB T1	WBC T1	*β*-Carotene T1	
Arg T1	1	-0.008	0.076	-0.036	0.010	Pearson coefficient
		0.933	0.440	0.719	0.920	*p* value (2-tailed)

PLT T1	-0.008	1	0.122	0.330^∗∗^	-0.194^∗^	Pearson coefficient
	0.933		0.215	0.001	0.047	*p* value (2-tailed)

ApoB T1	0.076	0.122	1	0.036	-0.029	Pearson coefficient
	0.440	0.215		0.716	0.770	*p* value (2-tailed)

WBC T1	-0.036	0.330^∗∗^	0.036	1	-0.236^∗^	Pearson coefficient
	0.719	0.001	0.716		0.015	*p* value (2-tailed)

*β*-Carotene T1	0.010	-0.194^∗^	-0.029	-0.236^∗^	1	Pearson coefficient
	0.920	0.047	0.770	0.015		*p* value (2-tailed)

^∗^, ^∗∗^, ^∗∗∗^Significance level (<0.05; <0.01; <0.001). Arg: arginine; ApoB: apolipoprotein B; PLT: platelets; T1: time point after three months of dietary supplementation with Flebotrofine®; WBC: white blood cells.

## Data Availability

The processed data supporting this observational study are discussed in the text and have been summarized in the tables presented in the manuscript in a clear and rational way. The authors think that the original raw data do not provide any additional information to what was discussed.
